# Supplementary material for: The causal relationship between the human gut microbiota and pyogenic arthritis: a Mendelian randomization study
Source: Front Cell Infect Microbiol. 2024 Nov 26;14:1452480. doi: 10.3389/fcimb.2024.1452480 (PMC11629706; doi:10.3389/fcimb.2024.1452480)
Supplement: Supplementary file 1 [file DataSheet1.zip › mendelian test/ebi-a-GCST90027629.csv_leave_one_out.pdf]

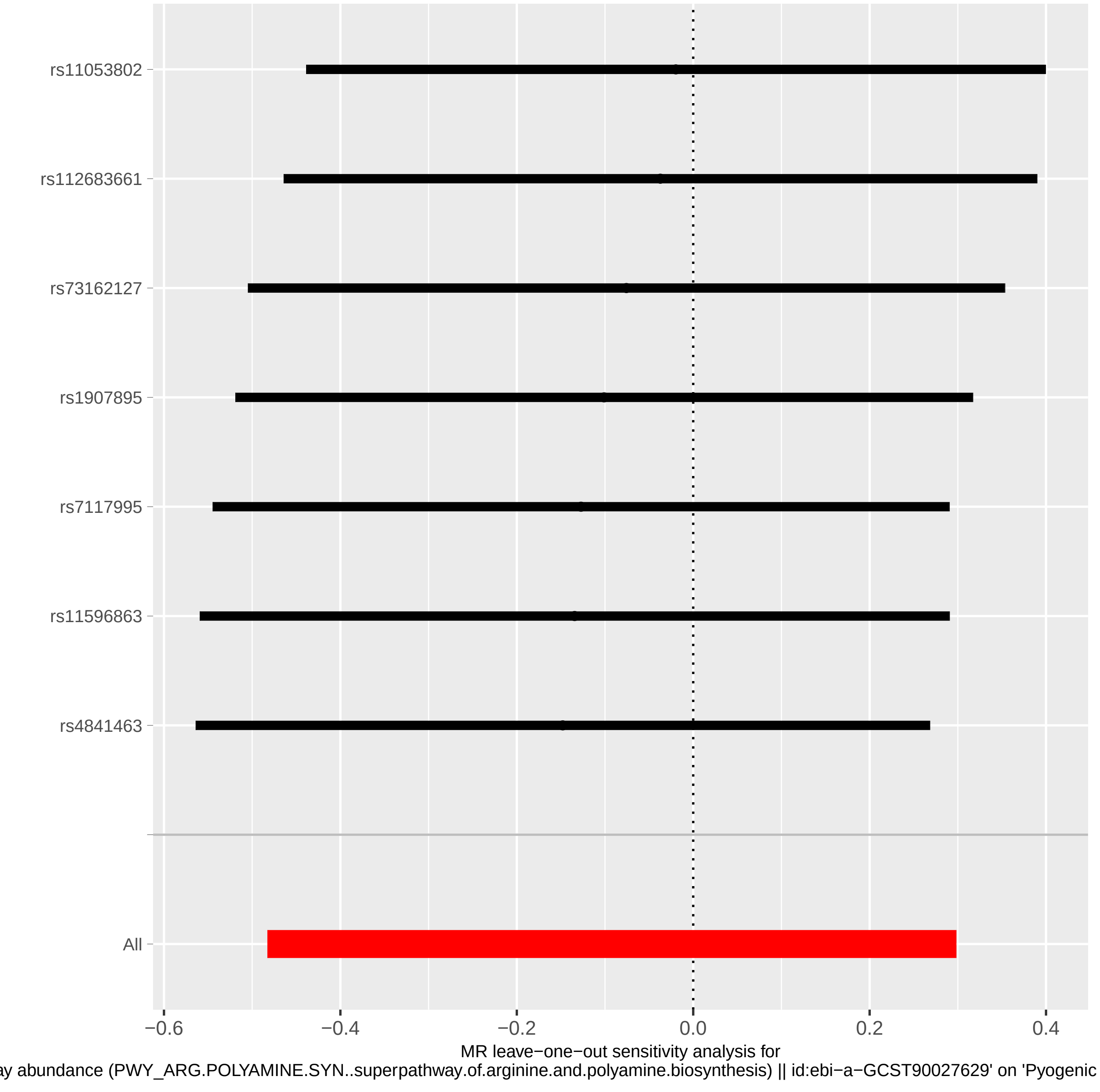
rs11053802
rs112683661
rs73162127
rs1907895
rs7117995
rs11596863
rs4841463
All
−0.6
−0.4
−0.2
0.0
0.2
0.4
MR leave−one−out sensitivity analysis for
ay abundance (PWY_ARG.POLYAMINE.SYN..superpathway.of.arginine.and.polyamine.biosynthesis) || id:ebi−a−GCST90027629' on 'Pyogenic
